# Genome-wide analysis reveals structured ecological and functional divergence within *Geobacillus stearothermophilus*

**DOI:** 10.1038/s41598-026-55928-5

**Published:** 2026-06-12

**Authors:** Seira Arakane, Yu Sato, Masanori Hashino, Shintaro Maeno

**Affiliations:** 1 Graduate School of Science and Technology for Innovation, Yamaguchi University, Yamaguchi, Japan; 2 Research Center for Thermotolerant Microbial Resources, Yamaguchi University, Yamaguchi, 753-8515 Japan; 3 Laboratory of Microbial Genomics, Pathogen Genomics Center, National Institute of Infectious Diseases, Japan Institute for Health Security, Tokyo, Japan

**Keywords:** *Geobacillus stearothermophilus*, Extremophiles, Phylogenetics, Comparative genomics, Genomic plasticity, Computational biology and bioinformatics, Ecology, Ecology, Evolution, Genetics, Microbiology

## Abstract

*Geobacillus stearothermophilus* is a thermophilic bacterium widely used in food sterilization and industrial processes. Although it has long been treated as a single, well-defined species, its internal genomic diversity has not been systematically evaluated. Here, we analyzed 36 strains using comparative genomics to clarify the structure of diversity within this species. Phylogenetic analyses consistently revealed two major genomic groups. Genome similarity measurements showed that most strains met current species-level criteria, yet clear internal differentiation was present. The two groups differed in ecological origin and genome composition. Strains associated with food-related environments tended to have smaller genomes and fewer metabolic genes, whereas strains from natural thermal habitats possessed larger genomes and broader metabolic capabilities, including genes for carbohydrate and fatty acid utilization. A small number of strains displayed intermediate positions, suggesting gradual diversification rather than sharp separation. Despite pronounced internal structuring, the strains remain within accepted species boundaries. These findings demonstrate that substantial ecological and functional divergence can accumulate within a single bacterial species. Our results provide a genomic framework for understanding intraspecific diversity in thermophilic bacteria and illustrate the importance of interpreting genome similarity thresholds in the context of population structure.

**Supplementary Information:**

The online version contains supplementary material available at 10.1038/s41598-026-55928-5.

## Introduction

Accurate classification of prokaryotes is essential for interpreting microbial diversity, ecology, and evolution^1,2^. Traditional taxonomy based on phenotypic traits often lacks resolution at the strain level, as these traits may vary within species and be influenced by environmental conditions^1,3^. Genome-based classification frameworks have transformed bacterial systematics, yet how these thresholds behave in structured populations remains insufficiently explored.

Thermophilic bacteria are of particular interest due to their adaptations to high-temperature environments and their industrial relevance^4,5^. The genus *Geobacillus*, reclassified from *Bacillus* based on 16S rRNA analyses, comprises thermophilic, Gram-positive, spore-forming species widely distributed in natural and industrial environments^4^. Among them, *Geobacillus stearothermophilus*, the type species of the genus, has long been regarded as a well-defined taxon and is extensively used in studies of thermal adaptation and food sterilization processes^4,6^.

Despite its taxonomic stability, considerable phenotypic variation has been reported among strains of *G. stearothermophilus*, including differences in motility, carbohydrate utilization, and growth temperature ranges^7–9^. Such phenotypic heterogeneity may reflect underlying genomic structuring within the species rather than simple intraspecific variation^4,10^.

With the widespread adoption of whole-genome sequencing, genome-based metrics such as average nucleotide identity (ANI) and digital DNA–DNA hybridization (dDDH) have become standard tools for species delineation. Although thresholds of ANI ≥ 95–96% and dDDH ≥ 70% are broadly accepted, borderline and discordant cases have increasingly been reported^2,11^. Previous comparative genomic studies of *G. stearothermophilus*, including ANI- and pangenome-based analyses, have suggested the presence of genomic heterogeneity among strains^8^. However, the relationship between phylogenomic structure, ecological origin, and genome-based species thresholds across a broader strain set remains incompletely understood.

Here, publicly available genome sequences of *G. stearothermophilus* were analyzed to characterize intraspecific genomic diversity and population structure. By integrating phylogenomic, similarity-based, and functional analyses, this study evaluates the extent and structure of genomic diversity within the species and examines how consistently ANI- and dDDH-based thresholds perform in a structured population. This study aims to understand how structured genomic variation accumulates within a nominally single bacterial species and what this implies for genome-based classification frameworks. Because genome-based thresholds are widely applied across bacterial systematics, cases in which different metrics yield discordant interpretations provide an opportunity to re-examine how species boundaries are operationally defined in structured populations.

## Materials and methods

### Draft genome sequencing and de novo assembly of five *G. stearothermophilus* strains

Five strains of *G. stearothermophilus*—JCM 11297, JCM 12216, JCM 12894, JCM 14450, and *Geobacillus* sp. JCM 30104—were newly sequenced in this study, as publicly available genome sequences were not available for these Japan Collection of Microorganisms (JCM) strains. All strains were cultivated in LB medium at 55 °C with shaking at 180 rpm. Total DNA was extracted using a phenol/chloroform method with glass beads, as previously described^12^. DNA libraries were prepared using the TruSeq DNA PCR-Free Library Prep Kit (Illumina) and sequenced on an Illumina NovaSeq platform, generating 151-bp paired-end reads. Approximately 0.98–1.83 Gb of sequence data were obtained for each strain, corresponding to an average sequencing coverage of ~ 150 × for all newly sequenced genomes. Adapter trimming was performed using Platanus_trim (version 1.0.3) with default settings, and de novo assembly was conducted using Platanus_B (version 1.2.6)^13^. Contigs shorter than 200 bp were discarded. Genome annotation was performed using the DFAST pipeline (version 1.2.6)^14^. Basic statistics of sequencing and assembly are summarized in Supplementary Table S1.

### Retrieval of genomic data of *Geobacillus* strains

All draft and complete genome sequences of *Geobacillus* species available in the NCBI RefSeq database as of December 2023 were retrieved. For the case of *G. thermoleovorans*, the type strain was not available in RefSeq and was therefore obtained from GenBank. When multiple assemblies were available for a single strain, only the complete genome sequence was retained. In addition to the five newly sequenced genomes, a total of 107 publicly available *Geobacillus* genomes were included in the analyses (Supplementary Table S1). Genome completeness and contamination were additionally assessed using CheckM2 (version 1.1.0) with the “predict” workflow^15^.

### Strain selection for comparative analysis

A total of 107 *Geobacillus* genomes were compiled for initial comparative analysis. Gene annotation was performed using DFAST (version 1.2.6)^14^. Genomes with low completeness or classified as *Parageobacillus* were excluded from downstream analyses. Two *Parageobacillus* species, *P. thermoglucosidasius* DSM 2542^T^ and *Parageobacillus toebii* DSM 14590^T^, were retained as outgroups. Based on the preliminary genome-wide phylogeny, 36 strains representing the diversity within the *G. stearothermophilus* clade were selected for detailed phylogenetic and comparative analyses.

### Phylogenetic analysis of *G. stearothermophilus* strains

As described above, two *Parageobacillus* species were used as outgroups for phylogenetic inference. Based on the preliminary genome-wide phylogeny, 36 strains representing the diversity within the *G. stearothermophilus* clade were selected for detailed phylogenetic and comparative analyses^16^. In parallel, an independent phylogenomic analysis was performed using GTDB-Tk (Genome Taxonomy Database Toolkit) version 2.4.0 with the classify_wf pipeline and GTDB reference database release R220 under default parameters. This workflow uses GTDB-defined marker genes within a standardized genome-based taxonomic framework.

For both analyses, *G. subterraneus* KCTC 3922ᵀ and *G. thermodenitrificans* DSM 465ᵀ were included as outgroups. Roary was primarily used for core genome phylogenetic reconstruction. Core gene detection and multiple sequence alignments were performed using Roary (version 3.13.0)^17^. Roary was run with a BLASTp identity threshold of 98% (-i 98). Core genes were defined as those present in 100% of strains. Maximum likelihood (ML) phylogenetic trees were constructed using RAxML (version 8.2.12) under the General Time-Reversible (GTR) model, with optimization via the Broyden–Fletcher–Goldfarb–Shanno (BFGS) algorithm and 100 bootstrap replicates, based on 633 core genes^18^. Phylogenomic analysis was also performed using UBCG2 (Up-to-date Bacterial Core Gene set v2), which extracts 81 conserved single-copy core genes. The analysis was run with default settings as described previously^19^, and the resulting concatenated alignment was used to infer a maximum-likelihood tree. All resulting phylogenetic trees were visualized using iTOL^20^.

### Genomic relatedness analysis using ANI and dDDH

To evaluate genome-based species boundaries within *G. stearothermophilus*, pairwise genomic similarity was assessed using average nucleotide identity (ANI) and digital DNA–DNA hybridization (dDDH). ANIb values were calculated using PyANI (version 0.2.7) based on BLAST + alignments^21^. FastANI (version 1.33) was additionally performed as a complementary approach for pairwise ANI estimation^22^. In parallel, pairwise dDDH values were obtained using the Genome-to-Genome Distance Calculator (GGDC) version 3.0 (http://ggdc.dsmz.de/distcalc2.php)^23^, applying formula 2 (identities/HSP length), which is recommended for draft genomes. ANI and dDDH matrices were subjected to hierarchical clustering using the hclust function (method = “average”) in R (version 4.3.3), as described previously^24^. To construct dendrograms, genomic distances were calculated as (100 − ANI) or (100 − dDDH), while the original similarity values were retained for all other comparative analyses. These analyses were performed following a previously described method with minor modifications^25^. To visualize the distribution of relatedness values, histograms of ANI and dDDH frequencies were generated for intra- and inter-cluster comparisons, as well as for the group of genomes. Bin widths were set to 0.1 for ANIb and 0.5 for dDDH, as described previously^26^.

### Functional annotation and identification of conserved and cluster-enriched genes

To investigate the functional potential and genomic differentiation between these clusters, multiple annotation and clustering approaches were employed.

Metabolic pathways were predicted using the KEGG Automatic Annotation Server (KAAS), which assigned KEGG Orthology (KO) numbers to the predicted coding sequences (CDSs) of each genome^27^. To estimate the conserved gene repertoire, all predicted protein sequences were grouped into orthologous clusters using GET_HOMOLOGUES (version 07112023), which applies all-against-all bidirectional BLAST comparisons and the MCL graph-based clustering algorithm^28^. GET_HOMOLOGUES was used for comparative gene content analyses and identification of cluster-enriched genes. Genes present in all analyzed genomes were defined as core genes. Cluster-enriched genes were defined as genes present in > 80% of strains in one cluster and < 20% in the other cluster. For comparative functional analysis between cluster 1 and cluster 2, the CDSs of each strain were assigned to functional categories using the eggNOG-mapper (version 2.1.12; http://eggnog-mapper.embl.de/) based on the clusters of Orthologous Groups (COG) classification^29^. Statistical significance of differences in genome size and gene counts between clusters was assessed using Welch’s *t*-test, as implemented in R (version 4.3.3). Statistical analyses were performed using Student’s t-test when variances were equal (F-test, p > 0.05) and Welch’s t-test otherwise. A value of *p* < 0.05 was considered statistically significant.

## Results

### Phylogenomic structure within the genus *Geobacillus* and identification of the *G. stearothermophilus* lineage

**Fig. 1:**
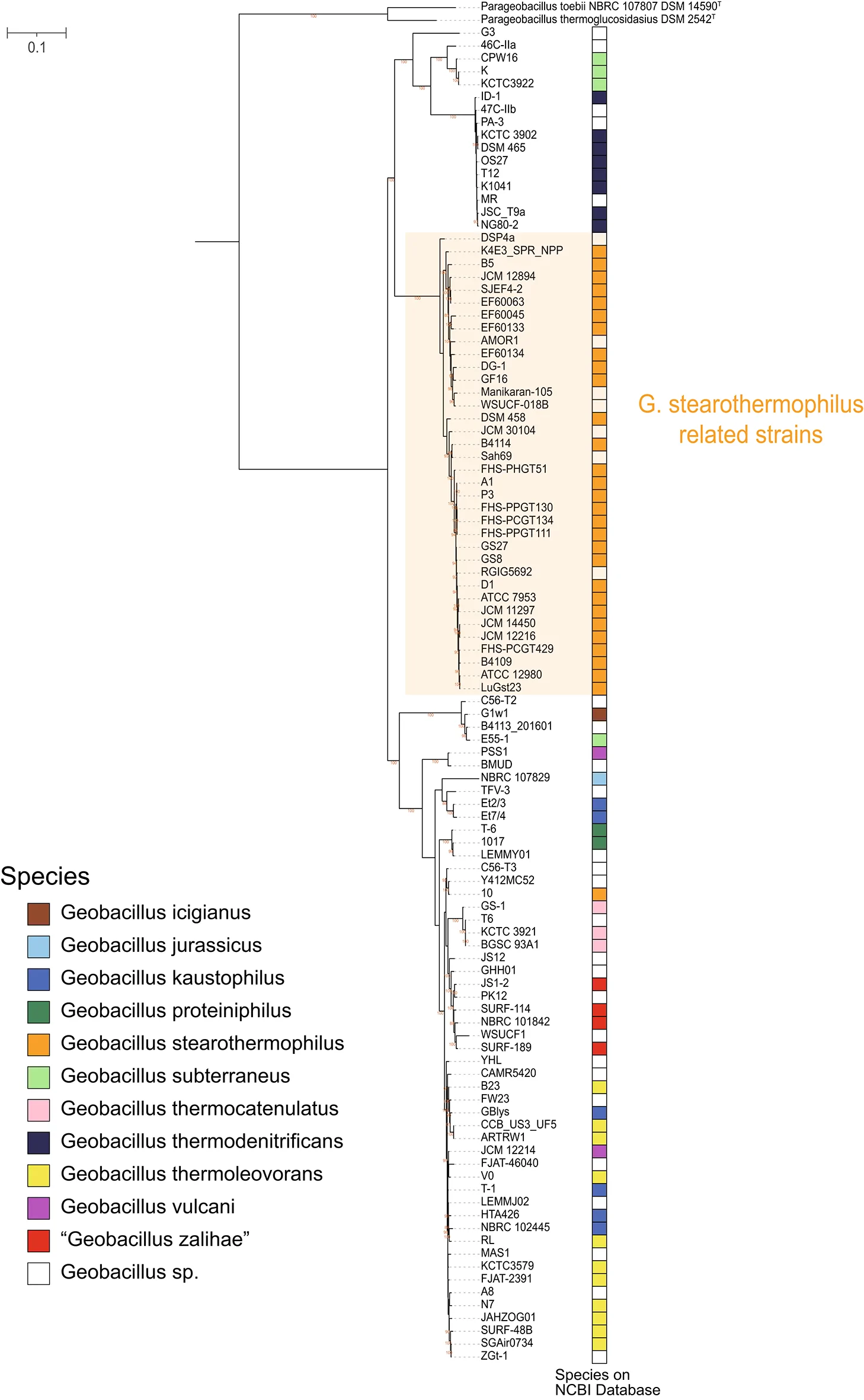
Phylogenomic tree of *Geobacillus* species inferred using UBCG2. The tree was reconstructed based on 81 conserved bacterial core genes using amino acid sequences. Numbers at nodes represent the Gene Support Index (GSI; maximum = 81). Bootstrap values (> 80%) based on 100 replicates are shown at branch nodes. *Parageobacillus toebii* DSM 14590^T^ and *Parageobacillus thermoglucosidasius* DSM 2542^T^ were used as outgroups. Scale bar, 0.1 substitutions per site.

The genus-wide phylogeny revealed limited interspecific resolution among several named species, particularly *G. kaustophilus* and *G. thermoleovorans*. In contrast, *G. stearothermophilus* formed a relatively well-supported clade that also included seven previously unclassified *Geobacillus* strains. The 36 strains were selected as members of the *G. stearothermophilus* lineage based on concordant placement in both GTDB-Tk classify_wf and UBCG2 phylogenomic analyses (Fig. 1). Strain JCM 30104 was reassigned to *G. stearothermophilus* based on phylogenomic analysis, whereas one strain originally labeled as G. stearothermophilus (strain 10) was reassigned to *G. thermoleovorans* according to GTDB-Tk classification. These findings underscore the blurred species boundaries within the genus. Several named *Geobacillus* species did not form clearly monophyletic groups in the genus-level phylogeny, suggesting that current taxonomy within the genus may not fully reflect genome-based evolutionary relationships. However, detailed genus-level taxonomic revision was beyond the scope of the present study.

### Phylogenetic structure of the *G. stearothermophilus* lineages

**Fig. 2:**
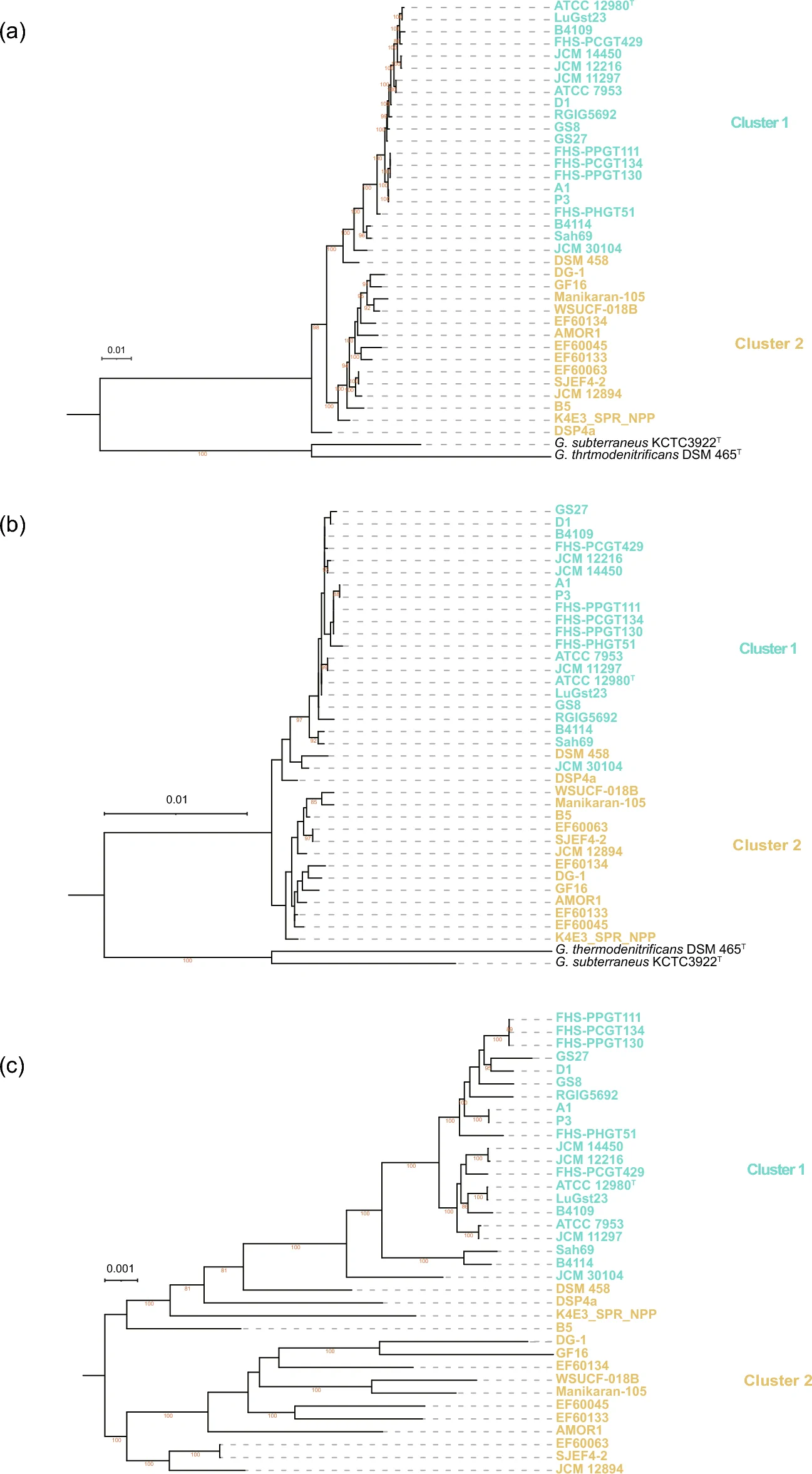
Phylogenomic reconstruction of 36 *Geobacillus stearothermophilus* strains using three independent approaches. Phylogenetic trees were inferred using (**a**) UBCG2 based on 81 bacterial core genes, (**b**) GTDB-Tk marker genes, and (**c**) a core genome alignment of 633 genes identified by Roary (BLASTp identity ≥ 98%) and reconstructed with RAxML. Across all three methods, strains consistently resolved into two major clades, demonstrating a robust bipartite structure within the species. Although minor topological differences were observed, the overall clustering pattern was conserved. Four strains (DSM 458, DSP4a, K4E3_SPR_NPP, and B5) exhibited variable placement near the boundary between clades. Two *Parageobacillus* strains were included as outgroups. Bootstrap values (> 80%, 100 replicates) are indicated at nodes.

To investigate the internal phylogenetic structure of the *G. stearothermophilus* lineage, 36 strains were analyzed using three independent genome-based approaches: UBCG2 (81 bacterial core genes), GTDB-Tk (GTDB-defined marker genes), and a Roary-based core genome phylogeny constructed from 633 core genes identified at ≥ 98% amino acid identity (Fig. 2a–c).

All three methods consistently resolved two major clades. One clade included the type strain ATCC 12980^T^ and was characterized by uniformly short internal branch lengths, whereas the second clade exhibited longer branches and greater internal divergence (Fig. 2a–c). Although minor differences in strain assignments were observed among methods, overall clade membership was highly concordant.

Four strains (DSM 458, DSP4a, K4E3_SPR_NPP, and B5) showed inconsistent placements across the three phylogenies and were located near the boundary between the two clades.

### Genome-based similarity and cluster concordance based on ANIb and dDDH

**Fig. 3:**
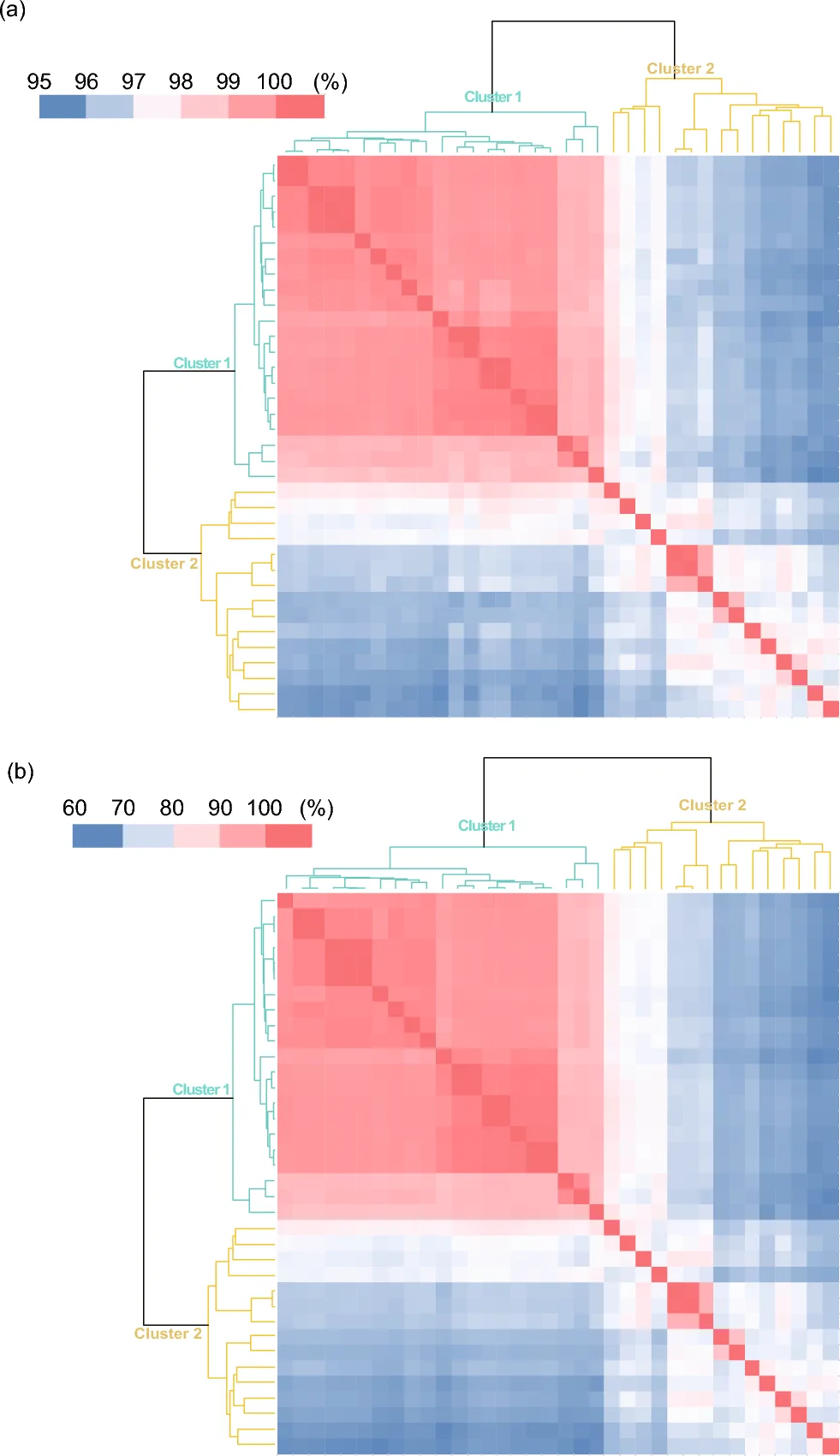
Heatmaps of pairwise genomic similarity among 36 *Geobacillus stearothermophilus* strains. (**a**) Average nucleotide identity (ANIb) and (**b**) digital DNA–DNA hybridization (dDDH) values were calculated for all pairwise strain comparisons. Hierarchical clustering based on similarity values revealed a consistent bipartite structure, with strains forming two major clusters. In the ANIb matrix, most pairwise comparisons exceeded the 95% species threshold, supporting assignment to a single species under conventional criteria. In contrast, the dDDH matrix displayed broader variability, with multiple inter-cluster comparisons falling below the 70% threshold, despite high ANIb values. Dendrogram branches indicate cluster 1 (green) and cluster 2 (yellow). The block-like structure of the heatmaps visually demonstrates structured intraspecific divergence within a nominally single species.

Pairwise genomic similarity among 36 strains was evaluated using ANIb and dDDH (Supplementary Tables S2, S3). The ANIb heatmap showed uniformly high similarity, with all values exceeding the 95% species threshold (Fig. 3a). In contrast, dDDH values were more variable (Fig. 3b); among inter-cluster comparisons, 59.9% fell below the 70% threshold, whereas 40.1% remained above it, indicating partial genomic separation without complete discontinuity.

**Fig. 4:**
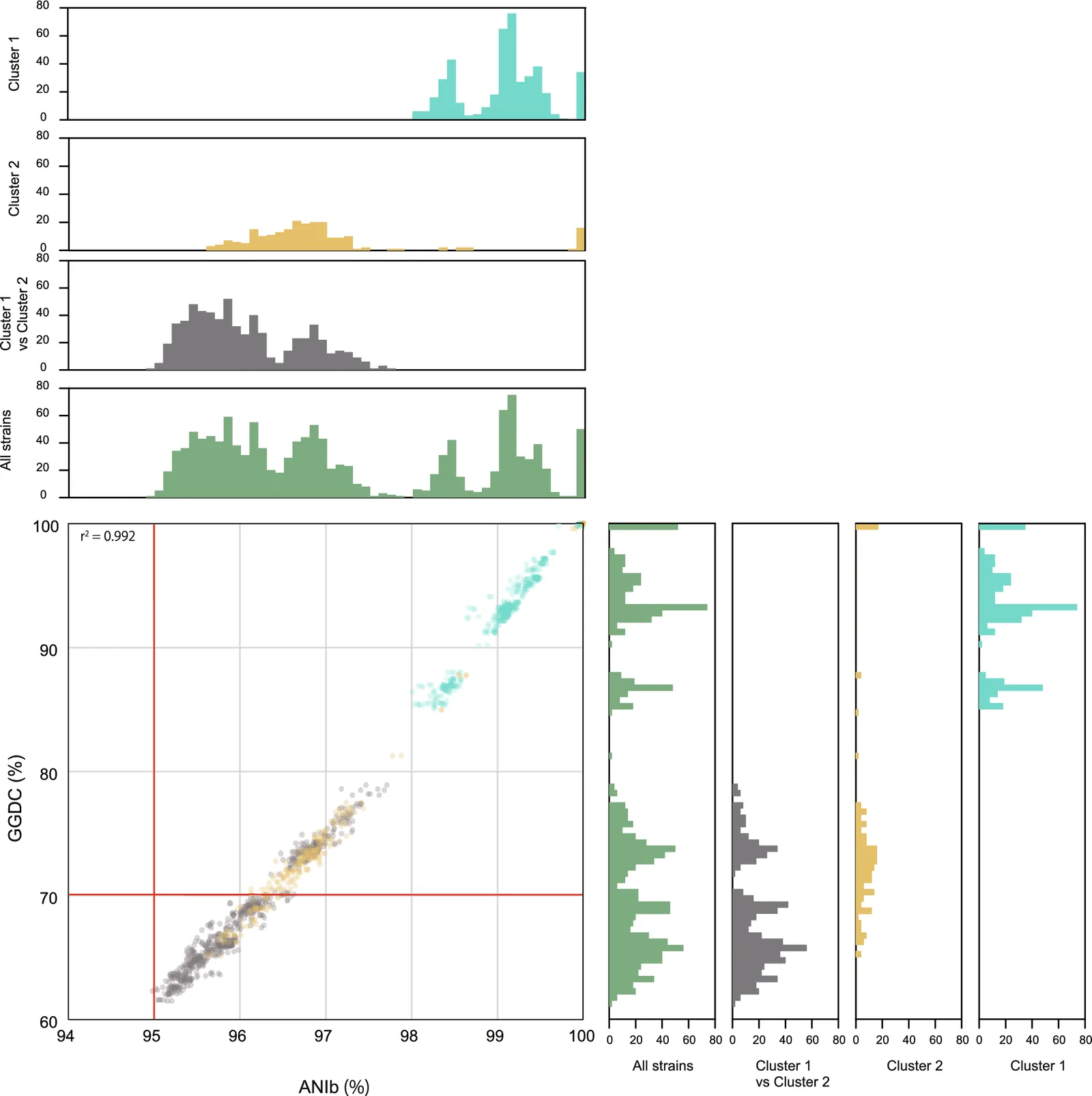
Distribution and correlation of genomic similarity metrics among *Geobacillus stearothermophilus* strains. Histograms display the distributions of ANIb (upper panels) and digital DNA–DNA hybridization (dDDH; middle panels) values for intra-cluster (cluster 1 and cluster 2) and inter-cluster strain comparisons. Cluster 1 exhibited a narrow and unimodal distribution, whereas cluster 2 showed broader and partially multimodal patterns, indicating higher internal genomic diversity. The lower panel shows the correlation between ANIb and dDDH values across all pairwise comparisons. Red lines indicate the commonly accepted species thresholds (ANIb = 95% and dDDH = 70%). Although a strong overall correlation was observed, dDDH values displayed greater dispersion near the species boundary. Notably, a substantial proportion of inter-cluster comparisons fell below the 70% dDDH threshold despite ANIb values exceeding 95%, illustrating metric-dependent interpretations of species boundaries.

Frequency distributions further illustrated this contrast (Fig. 4). ANIb values formed a narrow unimodal distribution, whereas dDDH values exhibited broader and partially multimodal patterns. Despite this variability, ANIb and dDDH were strongly correlated (r^2^ = 0.992), with increased dispersion at lower similarity levels.

**Fig. 5:**
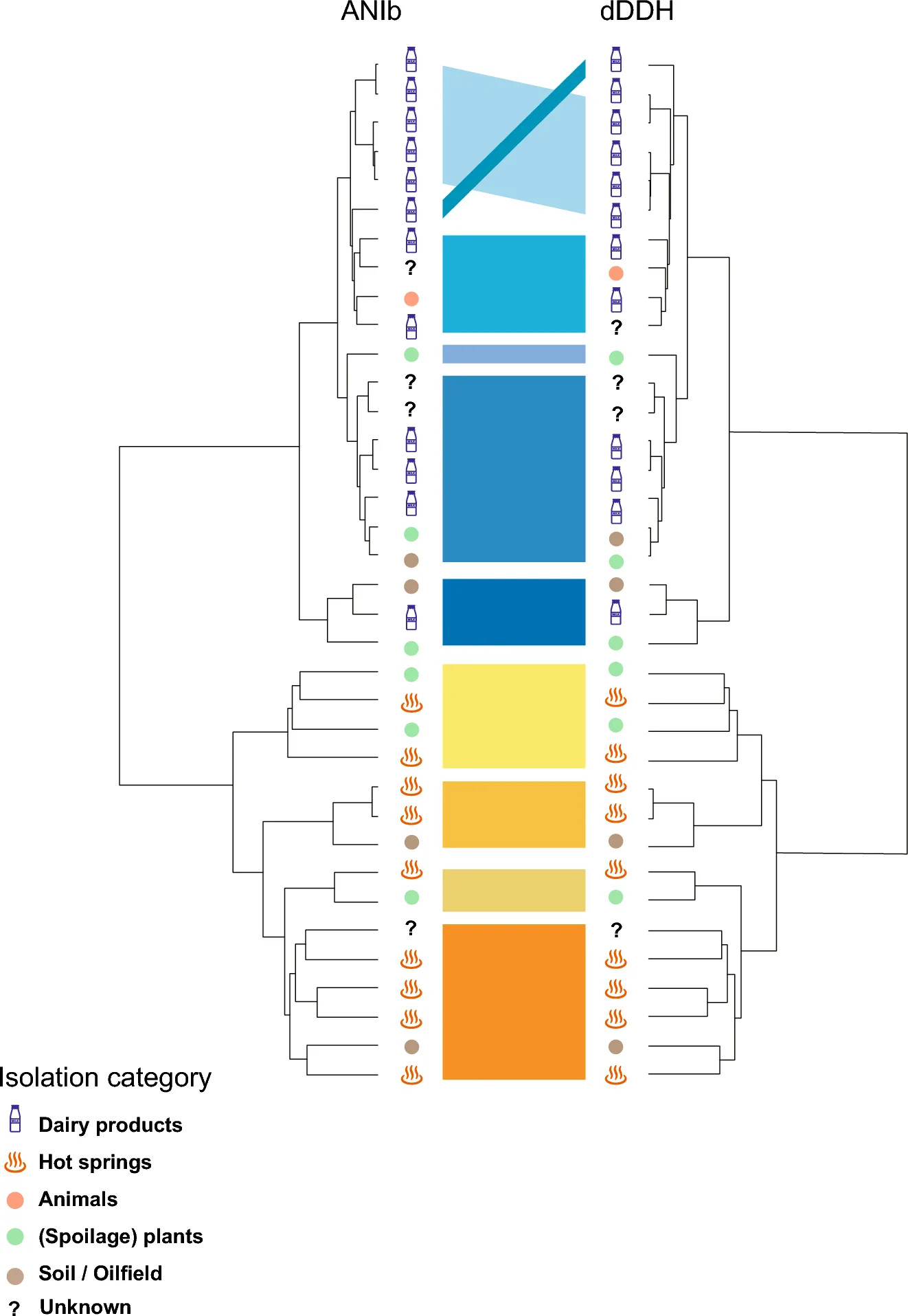
Hierarchical clustering of *Geobacillus stearothermophilus* strains based on genomic similarity. Dendrograms were constructed using hierarchical clustering (average linkage) of distance matrices derived from ANIb (100 − ANIb, left) and dDDH (100 − dDDH, right). In both analyses, strains were consistently separated into two major clusters, corroborating the bipartite structure observed in phylogenomic reconstructions. Isolation sources are indicated for each strain. A clear ecological pattern emerged, with one cluster predominantly associated with food-related environments and the other enriched in strains from natural thermal habitats. Strains with unknown isolation sources are labeled accordingly.

Hierarchical clustering based on (100 − ANIb) and (100 − dDDH) distances consistently resolved two major clusters with identical membership (Fig. 5). These two groups are hereafter referred to as cluster 1 and cluster 2. Cluster 1 (including the type strain ATCC 12980^T^) displayed high internal similarity (ANIb 98.0–100%; dDDH > 85%), whereas cluster 2 showed broader variation (ANIb 95.6–100%; dDDH 66–82%). These results support the presence of two robust genomic sublineages within *G. stearothermophilus*, with partial concordance to isolation source.

To evaluate the robustness of the observed genomic structure, additional ANI calculations were performed using FastANI. The resulting similarity patterns were highly consistent with those obtained using ANIb (Supplementary Fig. S1a). Additional comparisons using FastANI and GGDC produced highly correlated similarity patterns relative to ANIb-based analyses (Supplementary Fig. S1b), supporting the robustness of the observed bipartite genomic structure across different genome similarity metrics.

### Ecological origins and genomic features of each cluster

Isolation source metadata showed a clear ecological bias between the two clusters. Cluster 1 strains were predominantly isolated from food-associated environments, including powdered milk and dairy-related sources, whereas cluster 2 strains originated mainly from natural thermal habitats such as hot springs, oil reservoirs, and geothermal environments (Fig. 5, Supplementary Table S1). Several strains with ambiguous or unknown origins were distributed near the boundary between the two clusters.

**Fig. 6:**
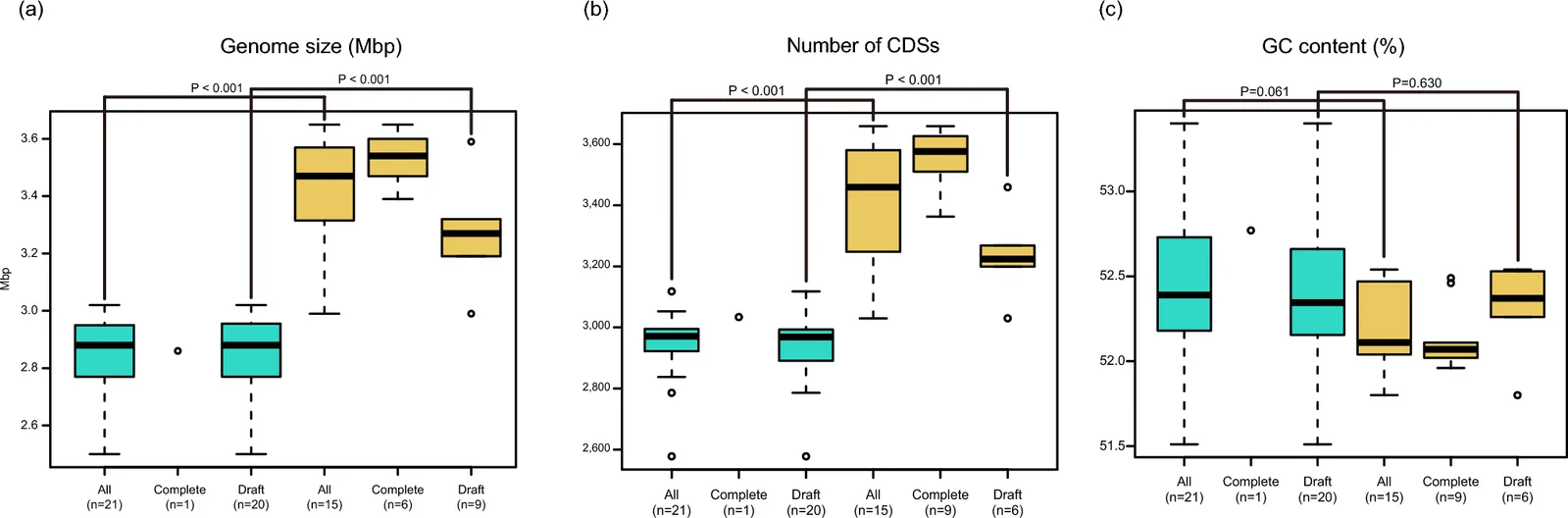
Comparison of genome characteristics between two phylogenomic clusters of *Geobacillus stearothermophilus*. (**a**) Genome size (Mbp), (**b**) number of coding DNA sequences (CDSs), and (**c**) GC content (%) were compared between cluster 1 and cluster 2. Boxplots are shown for all genomes, complete genomes only, and draft genomes only. Cluster 2 strains exhibited significantly larger genomes and higher numbers of CDSs than cluster 1 strains (two-sided Welch’s t-test, P < 0.001). No significant difference in GC content was detected. Sample sizes for each category are indicated below the plots.

Genome comparisons revealed clear differences in genome architecture between the clusters (Fig. 6a–c). Cluster 2 strains possessed significantly larger genomes (3.43 ± 0.18 Mbp) than cluster 1 strains (2.85 ± 0.13 Mbp; p < 0.001), accompanied by a higher number of predicted coding sequences (3,420 ± 187 vs. 2,940 ± 111; p < 0.001). These differences were maintained when analyses were restricted to draft genomes, indicating that assembly status did not bias the results.

In contrast, GC content did not differ significantly between clusters (~ 52.3% vs. ~ 52.5%; p > 0.05), suggesting that the observed divergence primarily reflects differences in genome size and gene content rather than overall nucleotide composition.

### Functional gene composition and metabolic differentiation between clusters

All predicted coding sequences were annotated using eggNOG-mapper and classified into 19 COG functional categories. Although the overall COG profiles were broadly similar between clusters, consistent quantitative shifts were observed (Table 1).

**Table 1:** Comparison of COG functional category distributions and cluster-specific genes between Cluster 1 and Cluster 2 of *Geobacillus stearothermophilus*.

COG category		Cluster 1			Cluster 2		
		Average of strains		Specific genes	Average of strains		Specific genes
		Average	SD		Average	SD	
C	Energy production and conversion	172	10	15	183	19	34
D	Cell cycle control, cell division, chromosome partitioning	65	4	0	57	4	2
E	Amino acid transport and metabolism	199	12	8	205	7	20
F	Nucleotide transport and metabolism	125	6	5	121	5	1
G	Carbohydrate transport and metabolism	112	7	8	126	19	20
H	Coenzyme transport and metabolism	128	6	3	125	3	7
I	Lipid transport and metabolism	54	6	14	72	5	33
J	Translation, ribosomal structure and biogenesis	194	18	0	176	7	1
K	Transcription	159	7	10	167	6	23
L	Replication, recombination and repair	255	44	3	290	66	1
M	Cell wall/membrane/envelope biogenesis	148	8	7	143	8	8
N	Cell motility	44	8	0	38	3	0
O	Posttranslational modification, protein turnover, chaperones	76	4	2	75	3	4
P	Inorganic ion transport and metabolism	163	10	4	170	7	16
Q	Secondary metabolites biosynthesis, transport and catabolism	17	5	10	18	2	15
S	Function unknown	825	37	21	821	24	46
T	Signal transduction mechanisms	81	5	6	83	6	11
U	Intracellular trafficking, secretion, and vesicular transport	31	5	0	31	6	0
V	Defense mechanisms	28	3	4	32	6	1

Cluster 1 showed relatively higher average gene counts in categories associated with translation ([J], p < 0.005), cell cycle control ([D], p < 0.005), nucleotide metabolism ([F], p < 0.005), and cell motility ([N], p < 0.005). In contrast, cluster 2 displayed higher gene counts in energy production ([C], p < 0.005), carbohydrate metabolism ([G], p < 0.005), lipid metabolism ([I], p < 0.005), transcription ([K], p < 0.005), and replication and repair ([L], p < 0.005). These differences suggest that cluster 2 genomes are generally expanded in metabolic and regulatory functions, whereas cluster 1 genomes show relative enrichment in core cellular processes.

KEGG pathway analysis further clarified the functional implications of these trends (Table 2). Cluster 2 strains encoded significantly more genes in central carbon metabolism (glycolysis, pentose phosphate pathway), oxidative phosphorylation, amino acid degradation, aromatic compound metabolism, and multiple transport systems (ABC transporters, two-component systems, and the phosphotransferase system). Particularly pronounced differences were observed in pentose and glucuronate interconversions and fatty acid degradation pathways. Correspondingly, key enzymes for D-xylose utilization (*xylA*, *xylB*) and β-oxidation (*fadA*, *fadN*) were present in nearly all cluster 2 genomes but absent from cluster 1.

**Table 2:** Comparison of representative KEGG metabolic pathways between *Geobacillus stearothermophilus* Cluster 1 and Cluster 2.

Pathway	Cluster 1 (n = 21)		Cluster 2 (n = 15)		P-value
	Average	SD	Average	SD	
Glycolysis / Gluconeogenesis (map00010)	29.5	1	34.1	2.3	< 0.05
Pentose phosphate pathway (map00030)	16.1	1.2	20.1	1	< 0.05
Pentose and glucuronate interconversions (map00040)	3.5	0.7	9.3	2.7	< 0.05
Fatty acid degradation (map00071)	6	0.3	11.9	1.4	< 0.05
Oxidative phosphorylation (map00190)	30.1	2.6	38	3.8	< 0.05
Valine, leucine and isoleucine degradation (map00280)	11.6	1.3	19	1.8	< 0.05
Lysine degradation (map00310)	3.1	0.5	6.9	1.1	< 0.05
Benzoate degradation (map00362)	3	0.2	7.5	0.9	< 0.05
Butanoate metabolism (map00650)	12.8	0.4	21.5	2.3	< 0.05
Sulfur metabolism (map00920)	11.9	0.5	15.7	1.9	< 0.05
ABC transporters (map02010)	59.6	3.1	76.8	5.7	< 0.05
Two-component system (map02020)	54.9	2.9	70.6	4.3	< 0.05
Phosphotransferase system (PTS) (map02060)	10.6	1.6	12.6	2.2	< 0.05

To further investigate gene repertoire differences between the two clusters, pan- and core-genome analyses were performed using GET_HOMOLOGUES. Pan- and core-genome analyses indicated substantial accessory genome diversity within the analyzed strains and supported an open pangenome structure (Supplementary Fig. S2). Cluster-enriched gene analysis supported these observations. Of 1,530 core genes shared by all strains, 131 genes were enriched in cluster 1 and 233 genes in cluster 2 (Supplementary Tables S4 and S5). Cluster 2-enriched genes were predominantly associated with expanded metabolic functions, including carbohydrate and lipid utilization and respiratory components (e.g., *nuo* subunits, *lctP*). In contrast, cluster 1-enriched genes were more frequently assigned to nucleotide metabolism [F], replication and repair [L], and defense mechanisms [V], consistent with a comparatively streamlined but growth-oriented genomic profile.

## Discussion

Genome-wide analyses revealed a reproducible bipartite structure within *Geobacillus stearothermophilus*. Across independent phylogenomic frameworks—including UBCG2, GTDB-Tk, core genome phylogeny, ANIb, and dDDH^30,31^—strains consistently grouped into two major clusters. This structured pattern coexisted with a borderline taxonomic scenario: while most ANIb values exceeded the 95% species threshold, dDDH values frequently approached or fell below the 70% cutoff^30,31^. The absence of clear discontinuity in ANI values, together with overlapping dDDH distributions and the presence of intermediate strains, supports continuous genomic differentiation rather than a discrete species boundary. Similar internal structuring has been documented in other bacterial taxa^32–34^, particularly in species with broad ecological distributions.

A clear association was observed between phylogenetic clustering and isolation source. Cluster 1 strains were predominantly derived from food-related environments, whereas cluster 2 strains originated mainly from natural thermal habitats such as hot springs and oil reservoirs^35^. Ecological structuring within bacterial species is often accompanied by adaptive divergence in gene content and genome organization^34,36,37^ and the present findings are consistent with this model.

The observed functional differences are also consistent with the ecological origins of the two clusters. Cluster 2 strains, which were mainly isolated from natural thermal environments such as hot springs and oil reservoirs, retained broader repertoires of genes related to carbohydrate utilization, fatty acid degradation, transport systems, and energy metabolism. Maintenance of expanded metabolic and transport capabilities has frequently been associated with adaptation to environmentally heterogeneous habitats, where nutrient availability fluctuates and broader substrate utilization provides ecological advantages^32,38^. In particular, the retention of genes involved in D-xylose utilization and β-oxidation in cluster 2 suggests the capacity to exploit more diverse carbon sources in natural thermal ecosystems.

In contrast, cluster 1 strains were predominantly isolated from food-associated environments and exhibited reduced genome sizes together with loss of several metabolic pathways. Similar patterns of genome reduction and functional specialization have been reported in bacteria adapted to relatively stable and nutrient-rich niches, where selective pressure to maintain broader metabolic versatility may be relaxed^39,40^. Although the ecological functions of these genes were not experimentally validated in the present study, the observed genomic patterns are consistent with niche-associated functional diversification within *G. stearothermophilus*.

Functional analyses further supported structured divergence. Cluster 2 genomes contained relatively higher gene counts in categories related to energy production (C), carbohydrate metabolism (G), lipid metabolism (I), and replication and repair (L), indicating broader metabolic capacity and environmental versatility. In contrast, cluster 1 genomes showed relative enrichment in translation (J), cell cycle control (D), nucleotide metabolism (F), and motility (N), suggesting a more streamlined and growth-oriented genomic profile. Importantly, these differences occur within overall genomic similarity ranges consistent with species-level classification, indicating functional diversification without taxonomic separation.

Four strains (DSM 458, DSP4a, K4E3_SPR_NPP, and B5) displayed inconsistent phylogenetic placement across analytical methods and occupied positions near the boundary between the two clusters. Their intermediate characteristics, including genome size and gene content, may reflect transitional or potentially recombining lineages rather than clearly delimited taxa^32,41–43^. The presence of such strains supports a continuum model of diversification within *G. stearothermophilus*, in which genomic divergence accumulates gradually and does not necessarily produce sharply defined boundaries.

Taken together, these findings indicate that *G. stearothermophilus* exhibits pronounced internal genomic structuring associated with ecological origin and functional divergence. However, the degree of divergence observed here does not unequivocally support formal species-level separation under current standards^30,31^. Rather, this case illustrates the limitations of applying uniform ANI and dDDH cutoffs to structured populations and highlights the importance of integrating phylogenomic context when interpreting genome-based similarity metrics.

## Conclusions

*G. stearothermophilus* exhibits pronounced internal genomic structure associated with ecological origin, genome content, and metabolic potential. Although two major phylogenomic clusters were consistently recovered across multiple analytical frameworks, most pairwise comparisons remained within currently accepted species boundaries. These findings indicate that substantial ecological and functional divergence can accumulate within a single bacterial species without necessarily producing discrete taxonomic separation.

The coexistence of high ANI values with more variable dDDH estimates further demonstrates that genome-based thresholds may behave inconsistently in structured populations. Rather than supporting immediate taxonomic revision, the present results highlight the importance of interpreting ANI and dDDH values within broader phylogenomic and ecological contexts. This study therefore provides a genome-based framework for understanding population-level diversification within thermophilic bacteria and underscores the need for nuanced application of genomic similarity thresholds in bacterial systematics.

## Supplementary Information

Supplementary Information 1.

Supplementary Information 2.

Supplementary Information 3.

Supplementary Information 4.

Supplementary Information 5.

Supplementary Information 6.

## Data Availability

The Whole Genome Shotgun projects of the G. stearothermophilus JCM 11297, JCM 12216, JCM 12894, and JCM 14450 strains, Geobacillus sp. JCM 30104 strain have been deposited in DDBJ/ENA/GenBank under the accession numbers BAAGGT010000000.1, BAAGGU010000000.1, BAAGGX010000000.1, BAAGGV010000000.1, and BAAGGW010000000.1, respectively.
