# Supplementary material for: Genome-wide analysis reveals structured ecological and functional divergence within Geobacillus stearothermophilus
Source: Sci Rep. 2026 Jun 12;16:26875. doi: 10.1038/s41598-026-55928-5 (PMC13519011; doi:10.1038/s41598-026-55928-5)

Supplementary Files

Supplementary Figure S1. Comparison of genome similarity estimates obtained using different ANI- and dDDH-based approaches.

(a) Correlation between ANIb values calculated using PyANI and FastANI values for pairwise genome comparisons among *G. stearothermophilus*. (b) Correlation between FastANI values and dDDH values calculated using GGDC formula 2. Each point represents a pairwise genome comparison. Dashed lines indicate linear regression fits, and the corresponding regression equations and coefficients of determination (R^2) are shown in each panel. Overall clustering patterns were highly consistent among methods, supporting the robustness of the observed bipartite population structure.

Figure S2. Pangenome and core-genome accumulation curves of the 36 *G. stearothermophilus* strains analyzed in this study.

The pangenome curve continued to increase with the addition of genomes, indicating an open pangenome structure ($\gamma = 0.321$). In contrast, the number of core genes gradually decreased and approached stabilization as additional genomes were included. Gene clustering was performed using GET_HOMOLOGUES.

Supplementary Figure S1. Correlation of ANIb, FastANI, and GGDC values among *G. stearothermophilus* strains

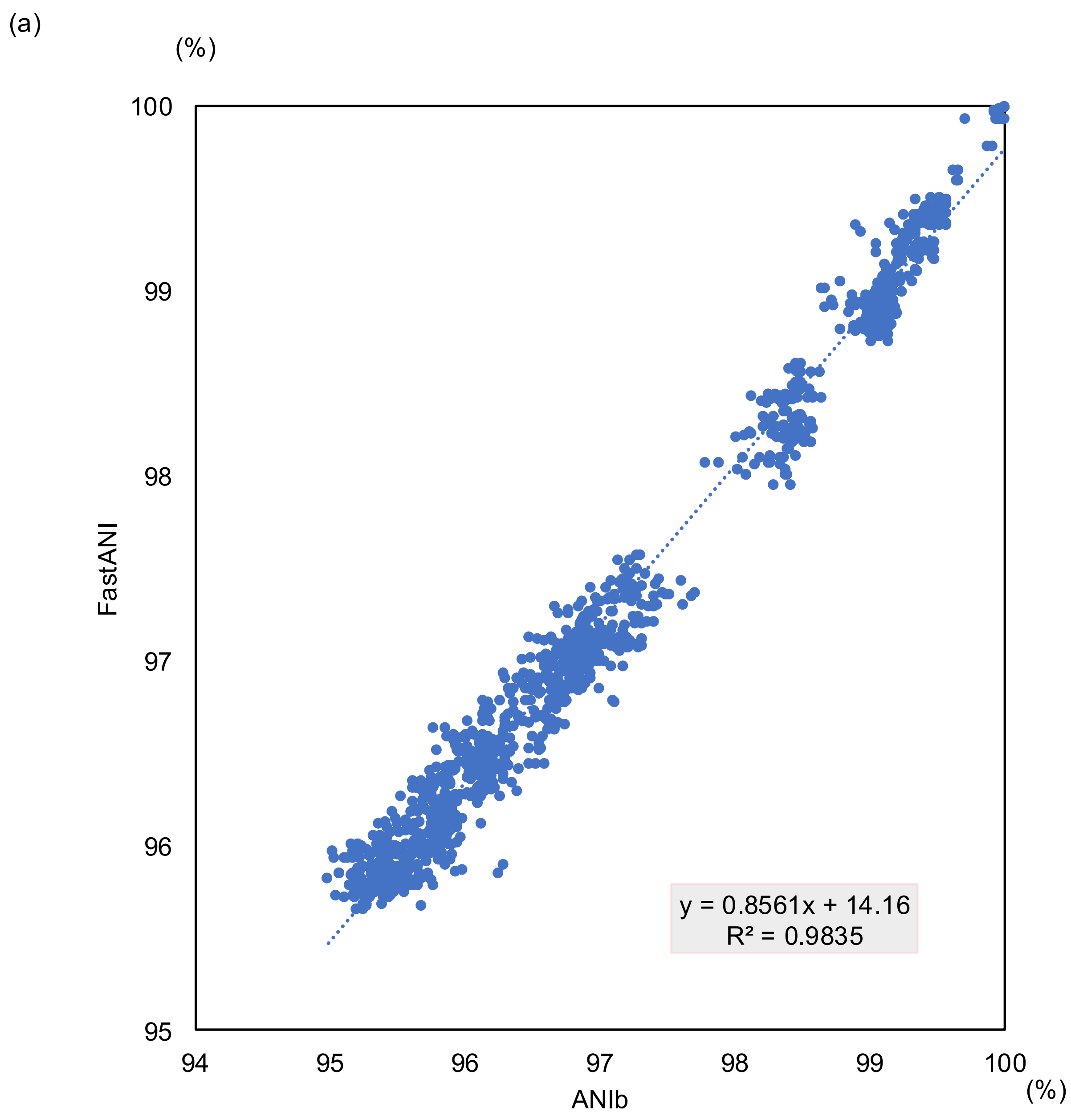

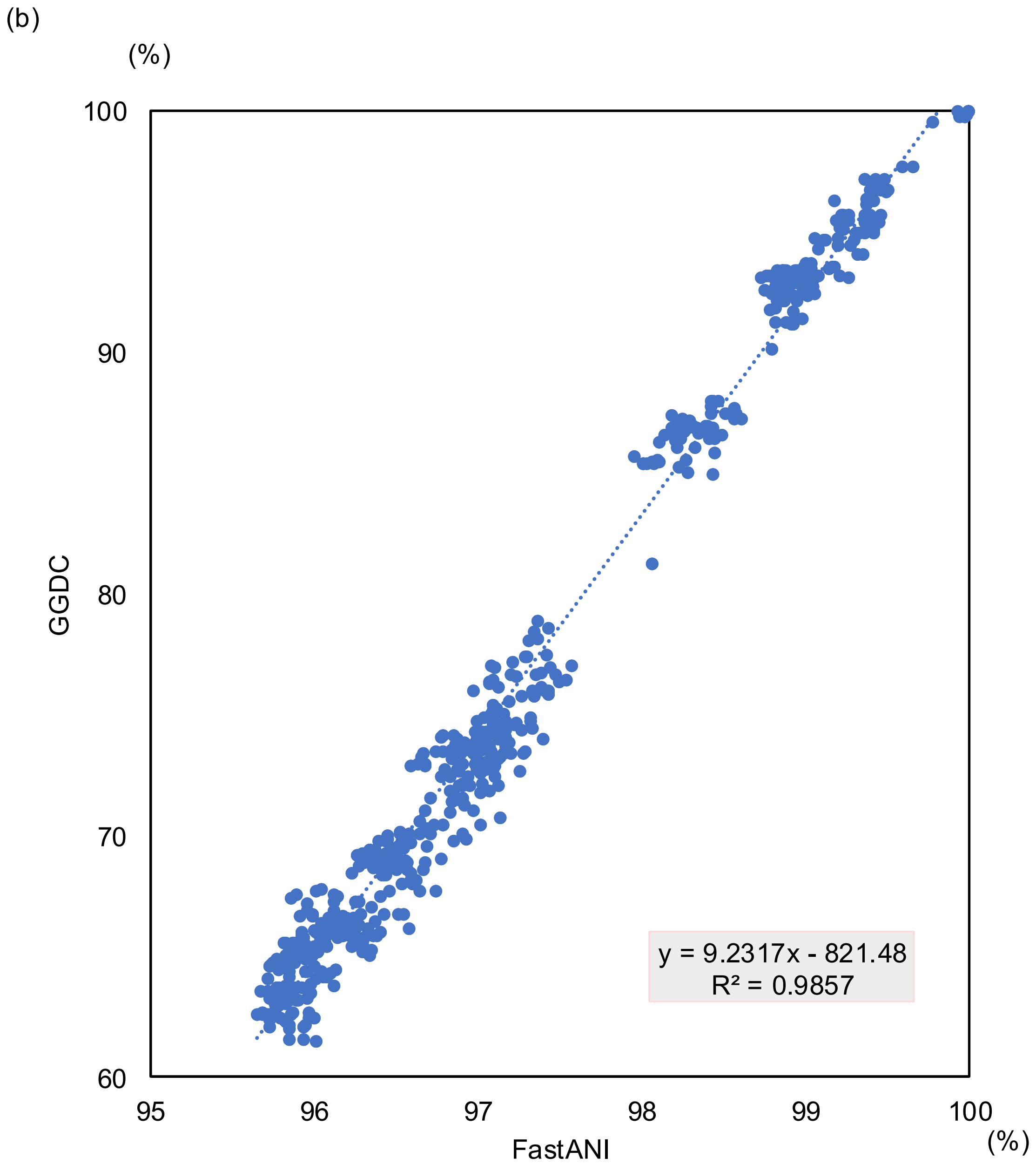

Supplementary Figure S2. Pangenome and core-genome accumulation curves of the 36 *G. stearothermophilus* strains analyzed in this study.

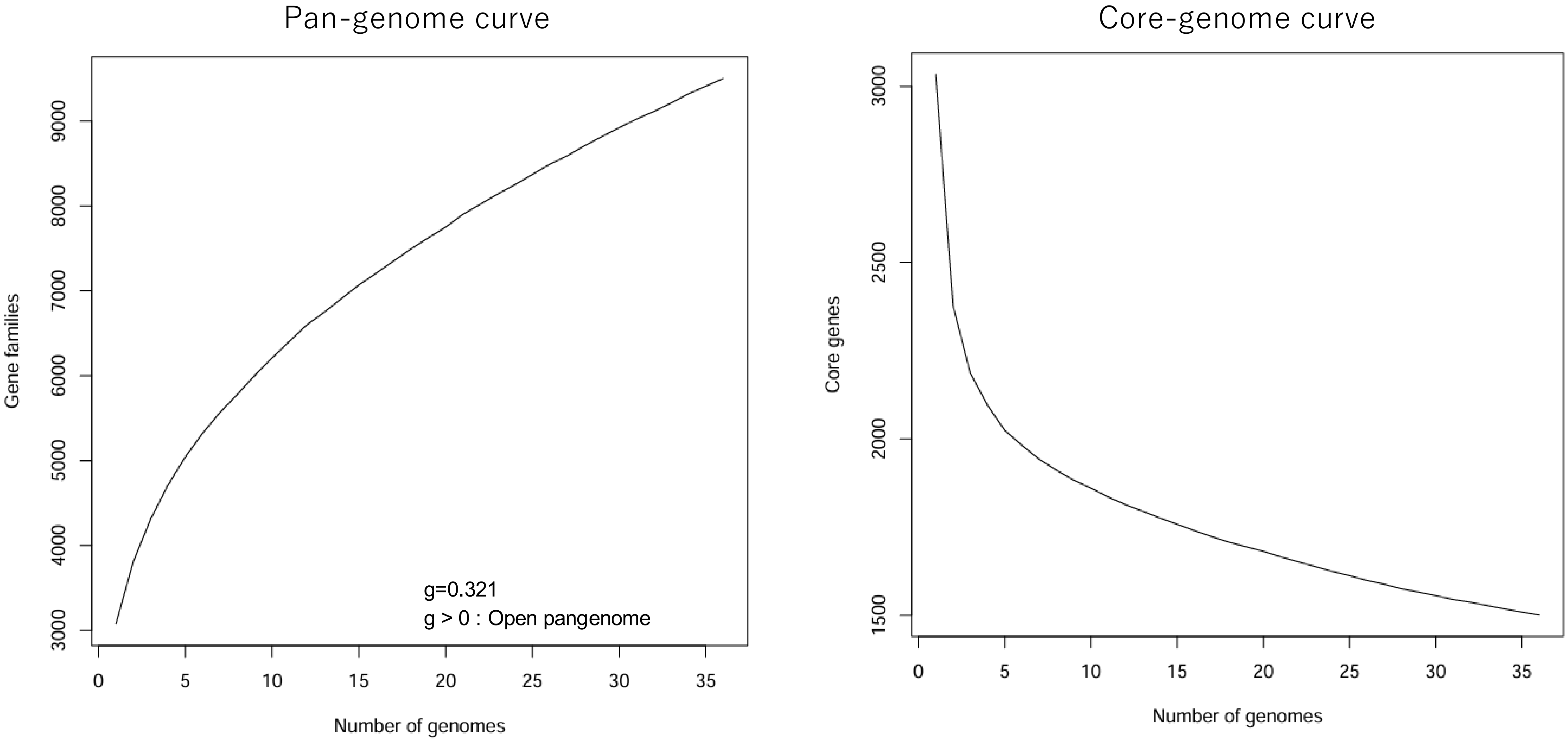

Supplement: Supplementary file 1 [file 41598_2026_55928_MOESM1_ESM.pdf]
